# Regulation of breast cancer oncogenesis by the cell of origin’s differentiation state

**DOI:** 10.18632/oncotarget.27783

**Published:** 2020-10-27

**Authors:** Sarah C. Petrova, Ihsaan Ahmad, Christine Nguyen, Stuart D. Ferrell, Sabrina R. Wilhelm, Yin Ye, Sanford H. Barsky

**Affiliations:** ^1^Cancer Center and Institute for Personalized Medicine, California University of Science and Medicine, Colton, CA 92324, USA; ^*^These authors contributed equally to this work

**Keywords:** transgenic mice, induced pluripotent stem cells (iPSCs), breast oncogenesis, differentiation

## Abstract

Human breast cancer which affects 1/8 women is rare at a cellular level. Even in the setting of germline BRCA1/BRCA2, which is present in all breast cells, solitary cancers or cancers arising at only several foci occur. The overwhelming majority of breast cells (10^9^–10^12^ cells) resist transformation. Our hypothesis to explain this rareness of transformation is that mammary oncogenesis is regulated by the cell of origin’s critical window of differentiation so that target cells outside of this window cannot transform. Our novel hypothesis differs from both the multi-hit theory of carcinogenesis and the stem/progenitor cell compartmental theory of tumorigenesis and utilizes two well established murine transgenic models of breast oncogenesis, the FVB/N-Tg (MMTV-PyVT)634Mul/J and the FVB-Tg (MMTV-ErbB2) NK1Mul/J. Tail vein fibroblasts from each of these transgenics were used to generate iPSCs. When select clones were injected into cleared mammary fat pads, but not into non-orthotopic sites of background mice, they exhibited mammary ontogenesis and oncogenesis with the expression of their respective transgenes. iPSC clones, when differentiated along different non-mammary lineages *in vitro*, were also not able to exhibit either mammary ontogenesis or oncogenesis *in vivo*. Therefore, *in vitro* and *in vivo* regulation of differentiation is an important determinant of breast cancer oncogenesis.

## INTRODUCTION

Cancers are common diseases in people and yet, on a cellular level, are quite rare [1–3]. The vast majority of both sporadic, spontaneous cancers and inherited germline cancers arise in single foci from singly transformed cells [4], despite the fact that, in the former, carcinogenic factors bathe fields of millions of potential target cells [3] and, in the latter, the predisposing germline mutations are present in every cell of the body [5, 6].

In the case of breast cancer, spontaneous, sporadic breast cancers are largely solitary in nature. Human breast cancers which arise from the effects of exogenous estrogen from hormone replacement therapy are also largely solitary. Even in the setting of inherited germline mutated BRCA1 or BRCA2, which is present in all the cells of the breast, only solitary cancers or multifocal cancers limited to 2 or 3 foci at most arise [7–9]. Attempts to explain the rareness of breast cancer at a cellular level have invoked the multi-hit theory of carcinogenesis, which basically opines that breast cancers do not occur unless there has been an accumulation of all of the necessary hits within the cell of origin [10, 11]. The multi-hit theory of breast carcinogenesis has also been invoked to explain such things as cancer latency, which is the period between cancer initiation and emergence and the cancer-aging relationship where an accumulation of “hits” over a period of time are necessary for cancer emergence. However, the multi-hit theory falls short in explaining the rareness of transformation at a cellular level [12–17]. This is so because the germline inherited BRCA1/BRCA2 breast cancers are caused by only 1 or 2 hits and certainly not multiple hits. And the external radiation, hormone replacement therapy, dietary carcinogens and pesticide exposure which cause the multi-hit spontaneous, sporadic breast cancers would be expected to bathe all the cells of the breast, subjecting them to all the “hits” required for carcinogenesis.

Another hypothesis that has been invoked to explain the rareness of transformation is the stem/progenitor cell compartmental theory of tumorigenesis [18–20]. That hypothesis opines that cancers including breast cancer contain a significant stem/progenitor cell compartment. The evidence for this belief is strong and multifaceted. For one, only the stem cell or progenitor cell subpopulation of a breast cancer is capable of self-renewal and multipotency. The proliferating subpopulation of a breast cancer is susceptible to radiation therapy and chemotherapy among other antiproliferative strategies. However, breast cancer stem/progenitor cells resist such antiproliferative strategies. Breast stem/progenitor cancer cells express different stem cell-associated genes, pathways and biomarkers of stemness that distinguish them from other tumor subpopulations. Breast cancer stem/progenitor cells are thought to be largely responsible for tumor relapses and recurrences [18–20]. Although tumor stem/progenitor cells represent a tumor compartment that is capable of self-renewal and multipotency, accounting for breast cancer relapses and recurrences, their presence does not account for the rareness at a cellular level of the *initial* transformation as the breast cancer stem/progenitor cell compartment is already transformed and considerable in size. In this study we advance a different and novel hypothesis to explain the rareness of breast cancer at a cellular level despite the very high incidence of the disease in women. Our hypothesis is that mammary oncogenesis is regulated by the cell of origin’s critical window of differentiation that is required for the *initial* transformation. Neither the multi-hit theory of carcinogenesis nor the stem/progenitor cell compartmental theory of tumorigenesis addresses or examines this critical window of differentiation.

In order to test our hypothesis this study utilized two well-known potent transgenic models of breast cancer, the FVB/N-Tg (MMTV-PyVT)634Mul/J and the FVB-Tg (MMTV-ErbB2) NK1Mul/J [21–23]. These models spontaneously develop breast cancer within the inguinal mammary fat pad at 60 days and 120 days after birth respectfully. Much of the oncogenic mechanism induced by the respective transgene has been elucidated in both of these models and interestingly, both transgenic mice exhibit multifocal breast cancer. Yet, the vast majority of the cells within their breasts do not transform. We reasoned that if we could obtain adult non-transformed cells from these transgenics and convert them to induced pluripotent stem cells (iPSCs) containing the oncogenic transgene, we could create a model where we could examine the effects of differentiation on both breast ontogenesis and oncogenesis and test our hypothesis.

## RESULTS

### Generation of iPSC clones

**Figure 1:**
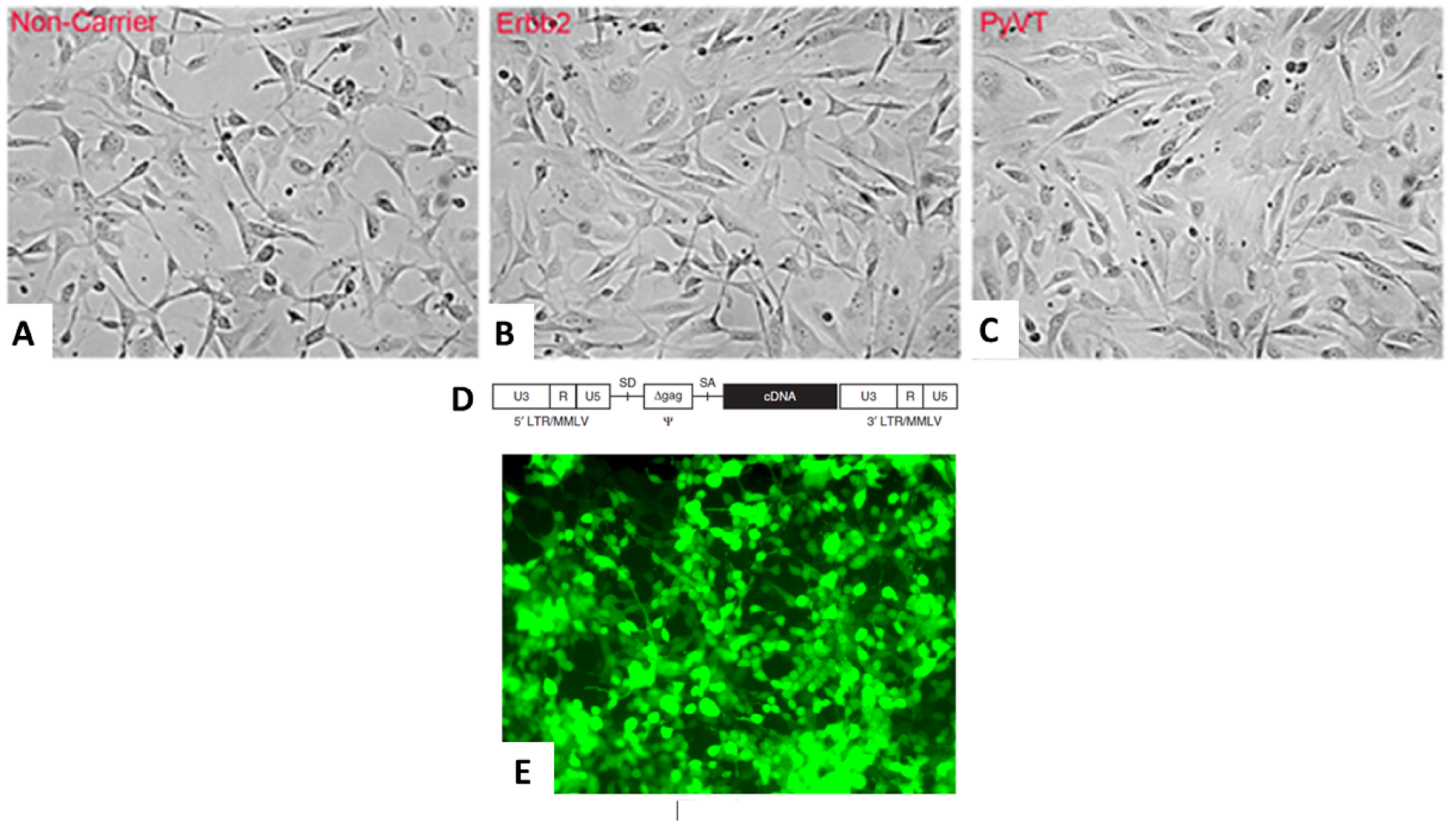
Retroviral transfection of tail vein fibroblasts. Two strains of transgenic and background mice were used to isolate and prepare tail vein fibroblasts. Noncarrier control FVB (**A**), FVB-Tg (MMTV-ErbB2) NK1Mul/ (**B**) and FVB/N-Tg (MMTV-PyVT)634Mul/J (**C**) generated identical appearing fibroblasts. pMXs-mSox2, pMXs-mOct3/4, pMXs-mKlf4, pMXs-mc-Myc and pMX-GFP were separately transfected into a Platinum A retroviral packaging cell line to produce retroviruses. The structure of the pMXs retroviral vector, both 5′- and 3′-long terminal repeats (LTRs), each consisting of U3, R and U5; and the ψ packaging signal consisting of a truncated gag sequence (Δgag) that exists between splicing donor (SD) and splicing acceptor (SA) is depicted (**D**). Platinum-A cells transfected with pMX-GFP exhibit green autofluorescence (**E**).

Tail vein fibroblasts could successfully be obtained from both female transgenics, the FVB/N-Tg (MMTV-PyVT)634Mul/J and the FVB-Tg (MMTV-ErbB2) NK1Mul/J and control noncarrier FVB mice. All the tail vein fibroblasts grew similarly (Figure 1A–1C). The tail vein fibroblasts were transfected with a cocktail of stem cell-inducing reporter genes, which were generated through a retroviral packaging cell line. Five plasmids with their respective inserts from Cell Biolabs Inc. were used to produce the following retroviruses: pMXs-mSox2, pMXs-mOct3/4, pMXs-mKlf4, pMXs-mc-Myc and pMX-GFP (Figure 1D). All these vectors were separately transfected into the Platinum-A Retroviral Packaging Cell Line, Amphotropic, to produce retroviruses expressing their respective stem cell or reporter gene (Figure 1E). The retroviruses obtained were equally mixed and transduced into the tail vein fibroblasts to induce iPSCs according to established methods [24].

**Figure 2:**
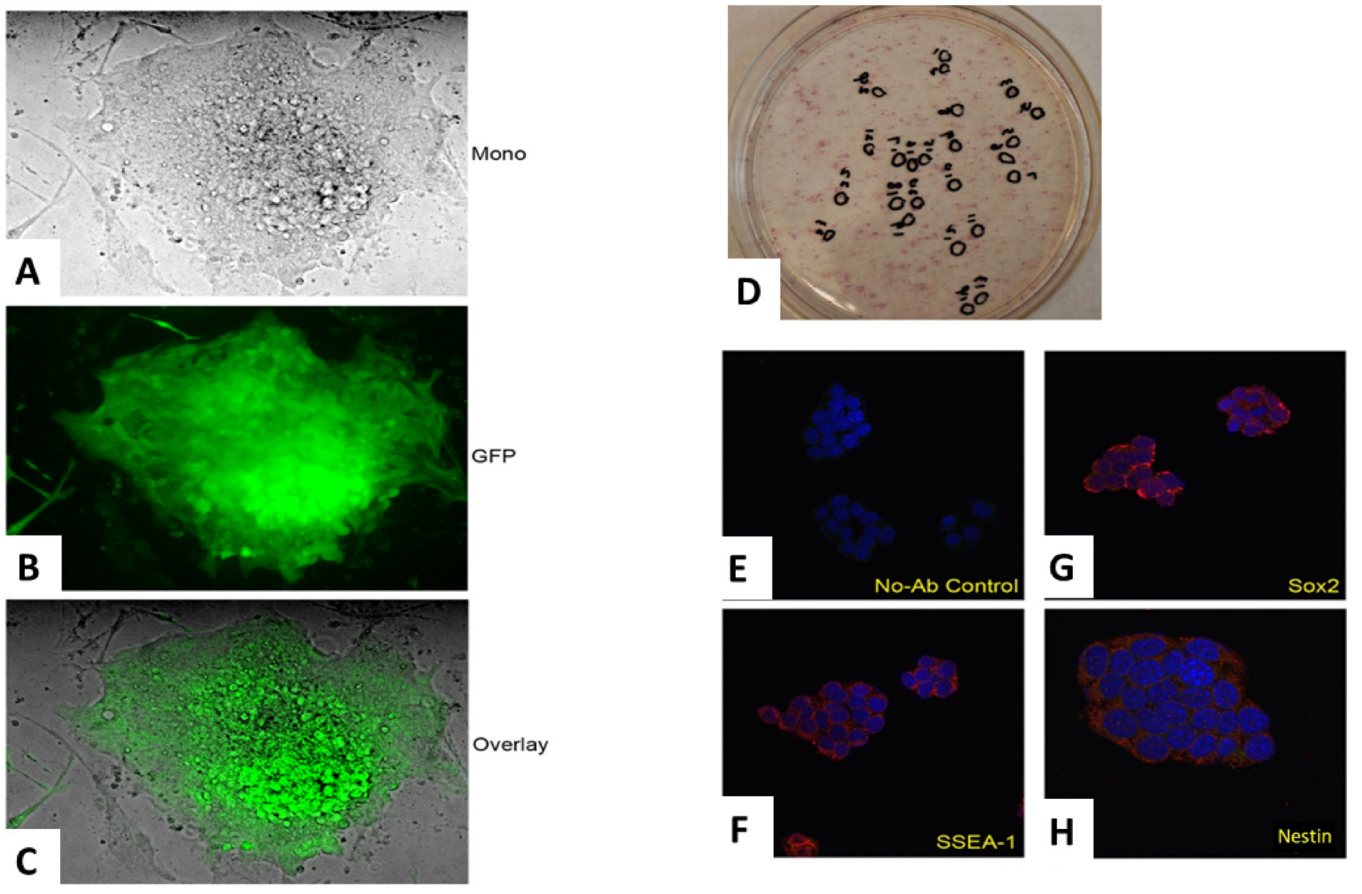
The production and confirmation of iPSC clones. The retroviruses from the preceding step were equally mixed and transduced into fibroblasts. Colonies became visible approximately 8–15 days after the retroviral infection. Morphology of an emerging colony of the monolayer by phase contrast (**A**), its GFP autofluorescence (**B**) and its merged overlay are depicted (**C**). Most emerging iPSC clones were positive for alkaline phosphatase, a known iPSC marker (**D**). Select iPSC clones exhibited other markers of pluripotent stem cells as illustrated by the following immunofluorescent studies: control (**E**), SSEA-1 (**F**), Sox-2 (**G**) and Nestin (**H**).

After two cycles of retroviral transduction, the transduced fibroblasts were cultured in embryonic stem (ES) medium (DMEM containing 15% FBS (vol/vol), 2 mM L-GIn, 1 × 10^-4^ M nonessential amino acids, 1 × 10^-4^ M 2-mercaptoethanol, 10 ng/mL LIF and 50 mg/mL of penicillin and streptomycin). Each day, the fibroblasts were fed fresh ES medium to generate iPSC clones. Approximately 20–30 clones emerged from each of the transduced fibroblast groups. Select single iPSC clones showing a characteristic 3D morphology (Figure 2A–2C) were selected and cultured in 24-well plates containing a SNL feeder layer.

### Identification and selection of iPSC clones

**Figure 3:**
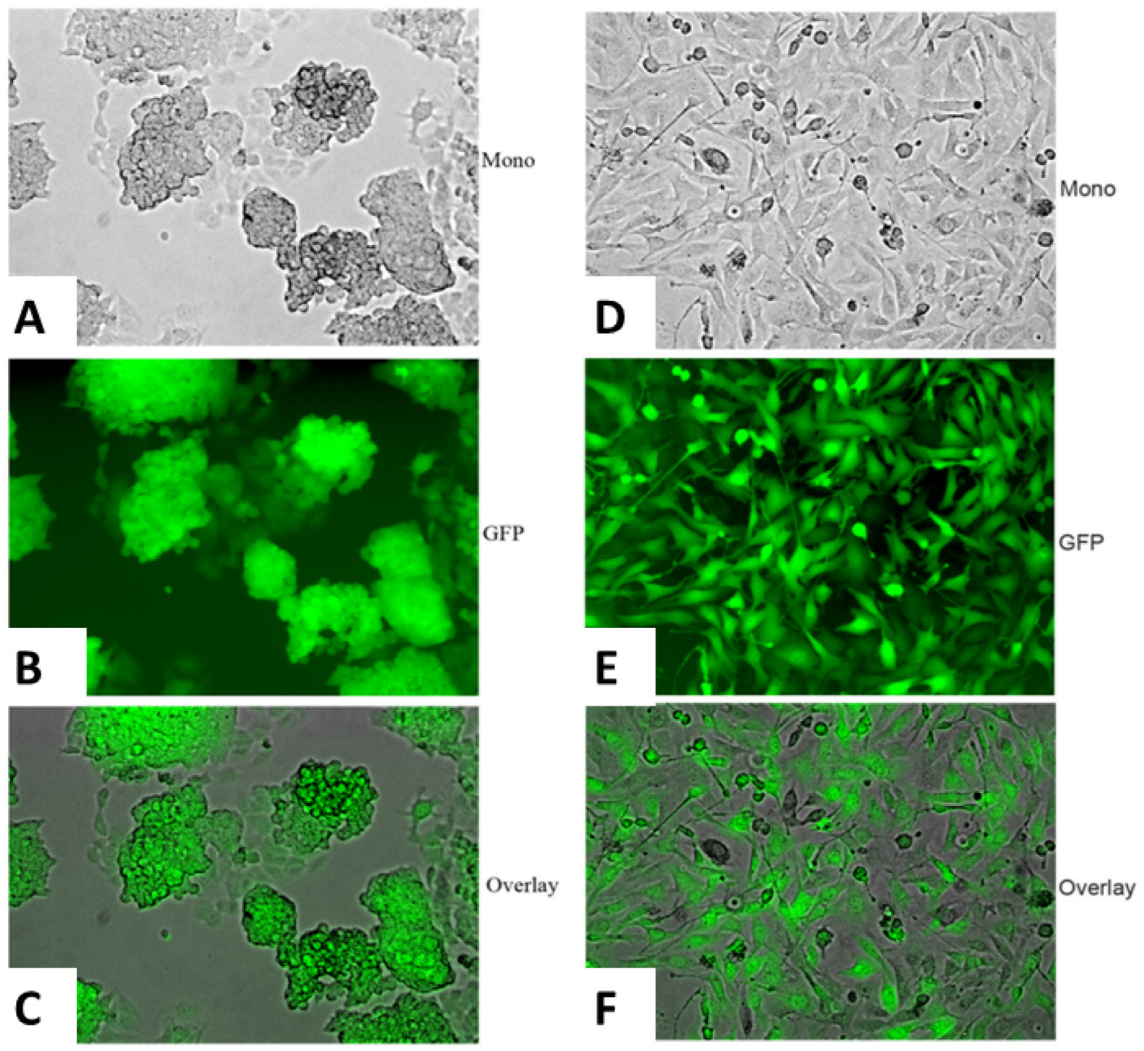
Heterogeneity of the iPSC clones. The emerging iPSC clones were heterogeneous in terms of morphology, growth rate and expression of the transfected genes: Sox, Oct3/4, Klf4 and c-Myc. Clone 1 was a typical iPSC clone as depicted by its monolayer by phase contrast (**A**), its GFP autofluorescence (**B**) and its merged overlay depicted (**C**). A representative clone that did not express all its transfected genes was more fibroblastic by its monolayer by phase contrast (**D**), its GFP autofluorescence (**E**) and its merged overlay depicted (**F**).

**Figure 4:**
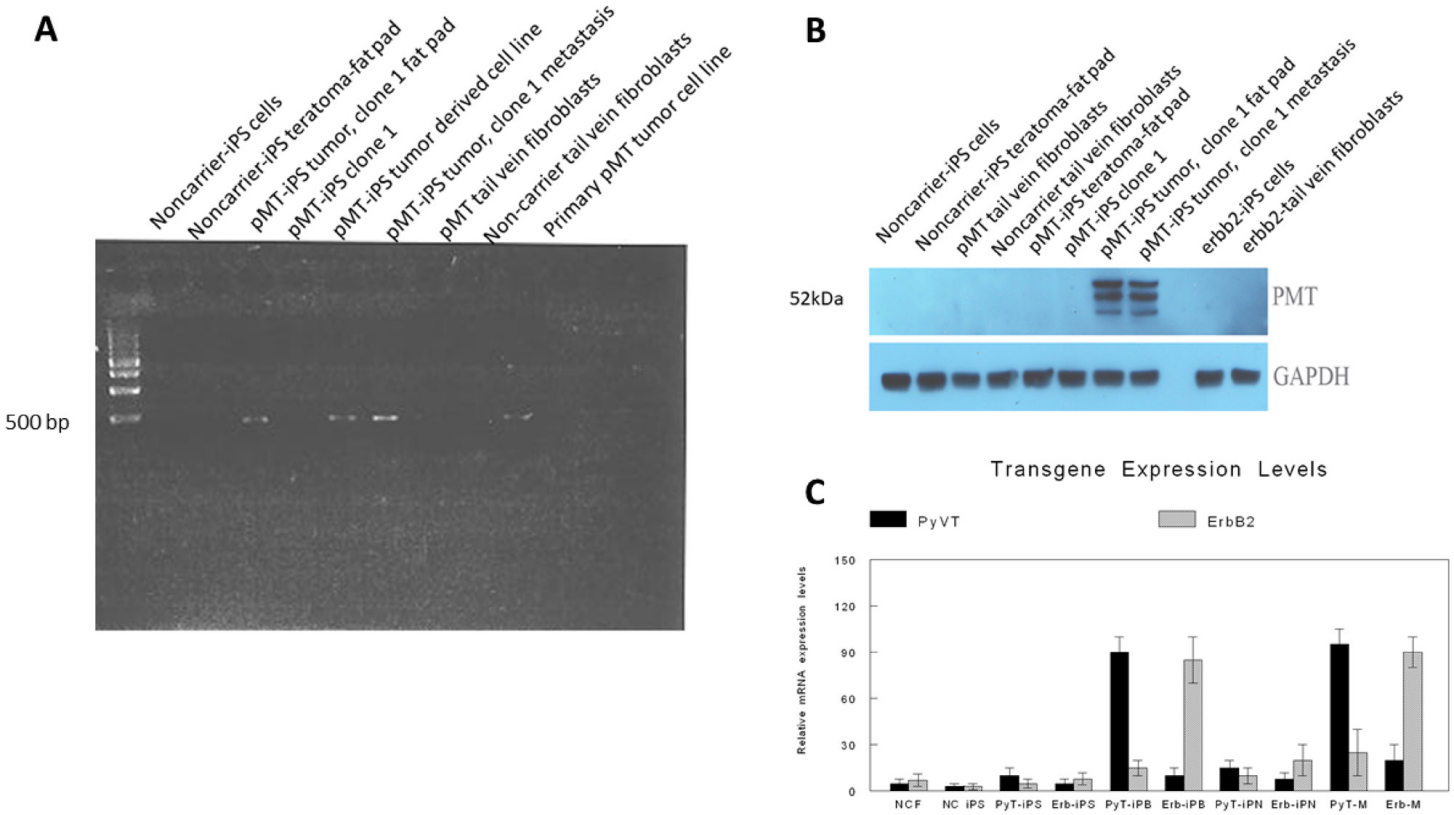
Expression of oncogenic transgenes. Expression of PyVT in various samples is depicted by RT-PCR (**A**) and Western blot (**B**). No PyVT transcripts nor protein was detected in noncarrier iPSCs, noncarrier teratomas arising in the fat pads, noncarrier tail vein fibroblasts, PyVT (pMT) tail vein fibroblasts, undifferentiated PyVT clones *in vitro*. PyVT transcripts and protein were detected in mammary carcinomas emerging from the PyVT clones injected in the mammary fat pad, metastases from the mammary carcinomas emerging from the PyVT clones, a derived cell line from the mammary carcinoma produced by the injected the PyVT clones and by the breast cancer arising in the original transgenic. Relative expression levels of both PyVT and ErbB2 is depicted by RT real time PCR (**C**) in the following samples: NCF, noncarrier tail vein fibroblasts; NC iPS, noncarrier iPSC clones; PyT-iPS, PyVT iPSC clones; Erb-iPS, ErbB2-iPSC clones; PyT-iPB, PyVT-iPSC clones injected into fat pad; Erb-iPB, ErbB2-iPSC clones injected into fat pad; PyT-iPN, PyVT-iPSC clones injected non-orthotopically; Erb-iPN, ErbB2-iPSC clones injected non-orthotopically; PyT-M, PyVT transgenic mouse; Erb-M, ErbB2-transgenic mouse.

Alkaline phosphatase, a known embryonal stem cell marker, was initially used to confirm the identity of the iPSC clones. The vast majority of the clones derived from the three groups of tail vein fibroblasts were indeed alkaline phosphatase positive (Figure 2D). After single clones were obtained, we used immunofluorescence to confirm the expression of known iPSC markers [24] including Sox2, Nestin and undifferentiated ES cell surface antigen, SSEA-1 (mouse-specific stage-specific embryonic antigen-1) (Figure 2E–2H). Other iPSC markers were also positive for select clones (data not shown). The iPSC clones obtained from each of the three groups of tail vein fibroblasts grew similarly and expressed identical iPSC markers overall. However, there was considerable heterogeneity within each group in terms of morphology, expression of stem cell markers, doubling time and expression of the respective transgene (Figure 3A–3F). Genotyping revealed the presence of the respective transgenes, PyVT and mutated ErbB2, in nearly all the iPSC clones derived from the two transgenics, FVB/N-Tg (MMTV-PyVT)634Mul/J and FVB-Tg (MMTV-ErbB2) NK1Mul/J, but predictably not from the noncarrier FVB background mice (data not shown). Clones that expressed all the expected iPSC markers, grew with typical iPSC morphology and genomically contained the respective transgene were selected for subsequent studies alongside the control noncarrier iPSC clones. The iPSC clones selected for study, however, did not express either transgene *in vitro* by either RT-PCR (Figure 4A), Western blot (Figure 4B) or RT-real time PCR (Figure 4C). Additionally, there was no expression of either transgene within these clones induced by dexamethasone *in vitro* (data not shown).

### Pluripotent differentiation of iPSC clones

**Figure 5:**
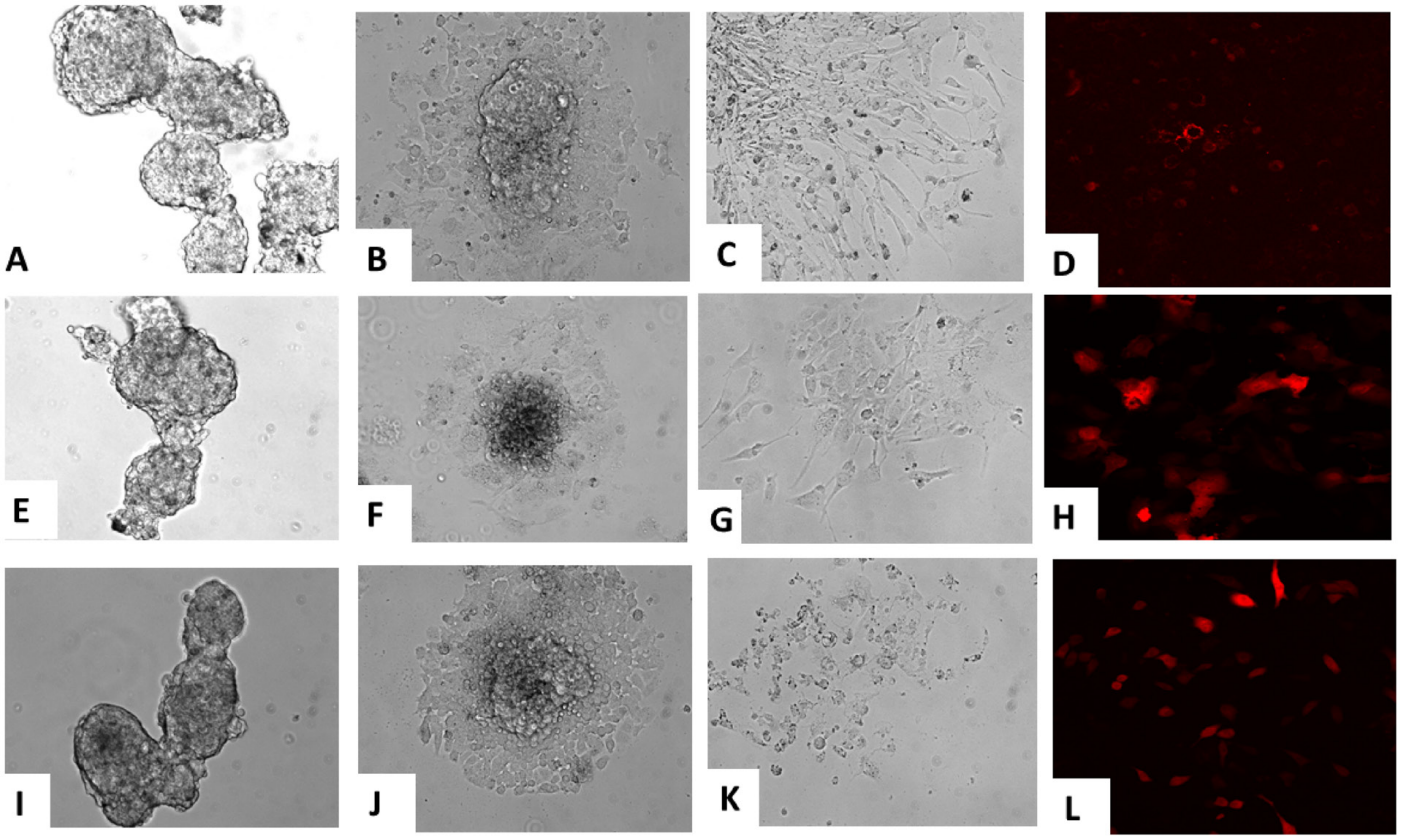
Pluripotent differentiation of iPSC clones *in vitro*. Differentiation of iPSCs into various lineages at different time points is depicted. Undifferentiated iPSC clones (**A**) were induced to differentiate into endothelial cells, depicted at day 7 (**B**) and then at day 15 (**C**). Similarly, undifferentiated iPSC clones (**E**) were induced to differentiate into hepatic cells, depicted at day 7 (**F**) and at day 15 (**G**). Additionally, undifferentiated iPSC clones (**I**) were induced to differentiate into osteocytes, depicted at day 7 (**J**) and at day 14 (**K**). Each of the lineage differentiations were confirmed by representative marker studies which included CD31 (endothelial) (**D**), albumin (hepatic) (**H**) and osteocalcin (**L**).

Select iPSC clones were differentiated into endothelial (Figure 5A–5D), hepatic (Figure 5E–5H) and osteogenic (Figure 5I–5L) lineages *in vitro* according to established methods [25–27]. Their morphology was monitored over 14 days. They first developed the morphology of embryoid bodies that continued to differentiate along the designated lineage. At the end of the differentiation period each lineage displayed specific biomarkers which included CD31 (endothelial) (Figure 5D), albumin (hepatic) (Figure 5H) and osteocalcin (osteogenic) (Figure 5L) through immunofluorescence studies. During this differentiation, none of the clones expressed the relevant oncogenic transgene (data not shown).

### Animal studies

#### Implantation studies

All animal studies were approved by University of Nevada’s School of Medicine and Nevada Cancer Institute’s IACUC, protocols 00439 and 00440. FVB female background mice were used to inject the selected iPSC and control clones. Clones were injected into the cleared inguinal mammary fat pads and into non-orthotopic subcutaneous sites. Mice were monitored over 8–16 weeks for mammary gland ontogenesis and oncogenesis.

#### Histological studies

**Figure 6:**
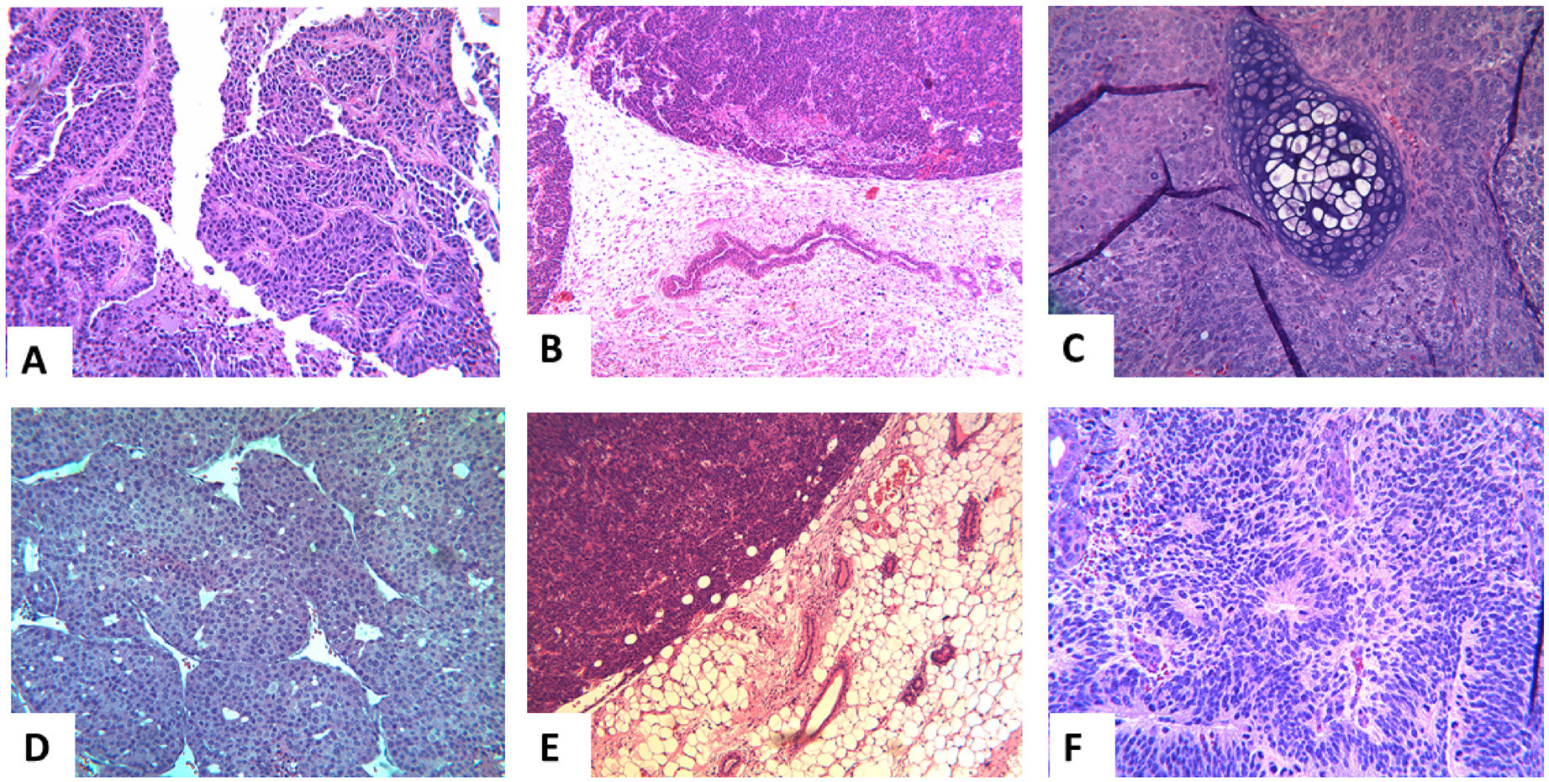
Representative histology of the extirpated tumors in the different groups. Representative histology of the transgenic breast cancers, the breast cancers produced by the transgenic-iPSCs and the teratomas produced by noncarrier iPSC clones and non-orthotopic transgenic-iPSC clones: breast cancer arising in the FVB/N-Tg (MMTV-PyVT)634Mul/J (**A**), from the fat pad of injected PyVT-iPSC clones (**B**), and a teratoma arising from an injected noncarrier iPSC clone (**C**). A similar pattern was observed in the ErbB2: breast cancer arising in the FVB-Tg (MMTV-ErbB2) NK1Mul/J (**D**), from the fat pad of injected ErbB2-iPSC clones (**E**) and a teratoma arising from an injected noncarrier iPSC clone (**F**). Teratomas were also observed with select PyVT-iPSC and ErbB2-iPSC clones when injected into non-orthotopic sites and occasionally in orthotopic sites as well.

The iPSCs derived from FVB/N-Tg (MMTV-PyVT)634Mul/J and FVB-Tg (MMTV-ErbB2) NK1Mul/J transgenics, when injected into the cleared inguinal mammary fat pads of background FVB mice, after approximately 60 days and 120 days respectfully, developed tumors. Emerging tumors were studied by routine light microscopy. The breast cancers arising in the PyVT transgenic (Figure 6A) strongly resembled breast cancers arising from the PyVT-iPSC clones injected into the mammary fat pad (Figure 6B). Occasionally, both noncarrier iPSC clones when injected orthotopically, and PyVT-iPSC clones when injected non-orthotopically, gave rise to teratomas (Figure 6C). The breast cancers arising in the ErbB2 transgenic (Figure 6D) also strongly resembled breast cancers arising from ErbB2-iPSC clones injected into the mammary fat pad (Figure 6E). Occasionally ErbB2-iPSC clones when injected non-orthotopically also gave rise to teratomas (Figure 6F).

### Fluorescence and immunocytochemical studies

**Figure 7:**
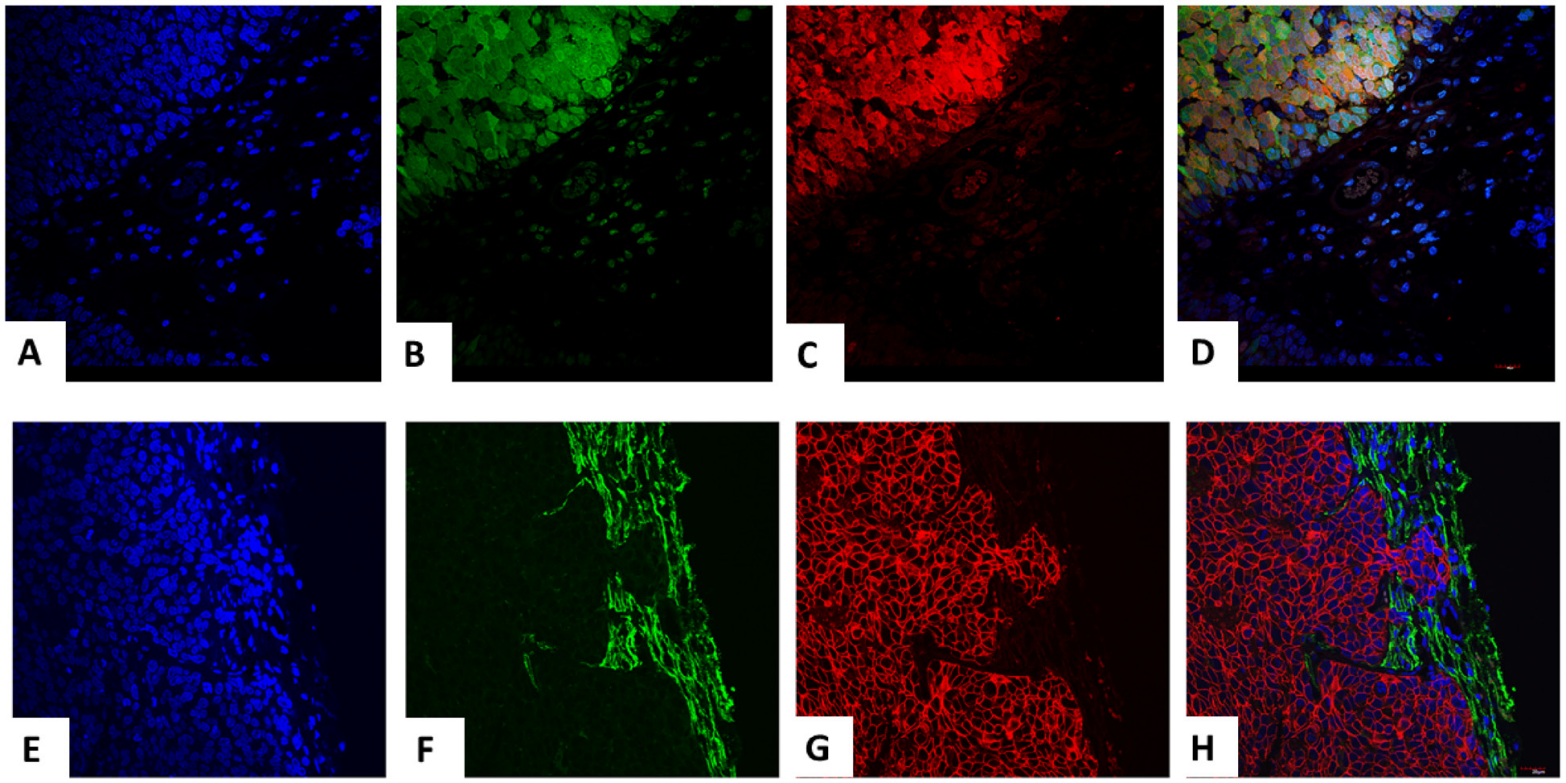
Trifluorescence studies of extirpated tumors in the transgenic-IPSC clones. Triple fluorescence studies on PyVT-iPSC generated extirpated tumors with DAPI blue nuclear autofluorescence (**A**), GFP green autofluorescence (**B**), Alexa Fluor^®^ 594 red immunofluorescence using goat anti-rat added to rat monoclonal to PyVT antigen (**C**) and its merged overlay depicted (**D**). Strong cytoplasmic expression of the PyVT antigen is observed. Similarly, triple fluorescence studies on ErbB2-iPSC generated extirpated tumors with DAPI blue nuclear autofluorescence (**E**), GFP green autofluorescence (**F**), Alexa Fluor^®^ 594 red immunofluorescence using goat anti-rabbit added to rabbit polyclonal antibody to ErbB2 (**G**) and its merged overlay depicted (**H**). Strong membrane expression of the ErbB2 antigen is observed.

PyVT-iPSC clones gave rise to mammary carcinomas that exhibited dual GFP autofluorescence and PyVT cytoplasmic red immunofluorescence (Figure 7A–7D). ErbB2-iPSC clones also gave rise to mammary carcinomas that exhibited dual GFP autofluorescence and ErbB2 membrane red immunofluorescence (Figure 7E–7H).

**Figure 8:**
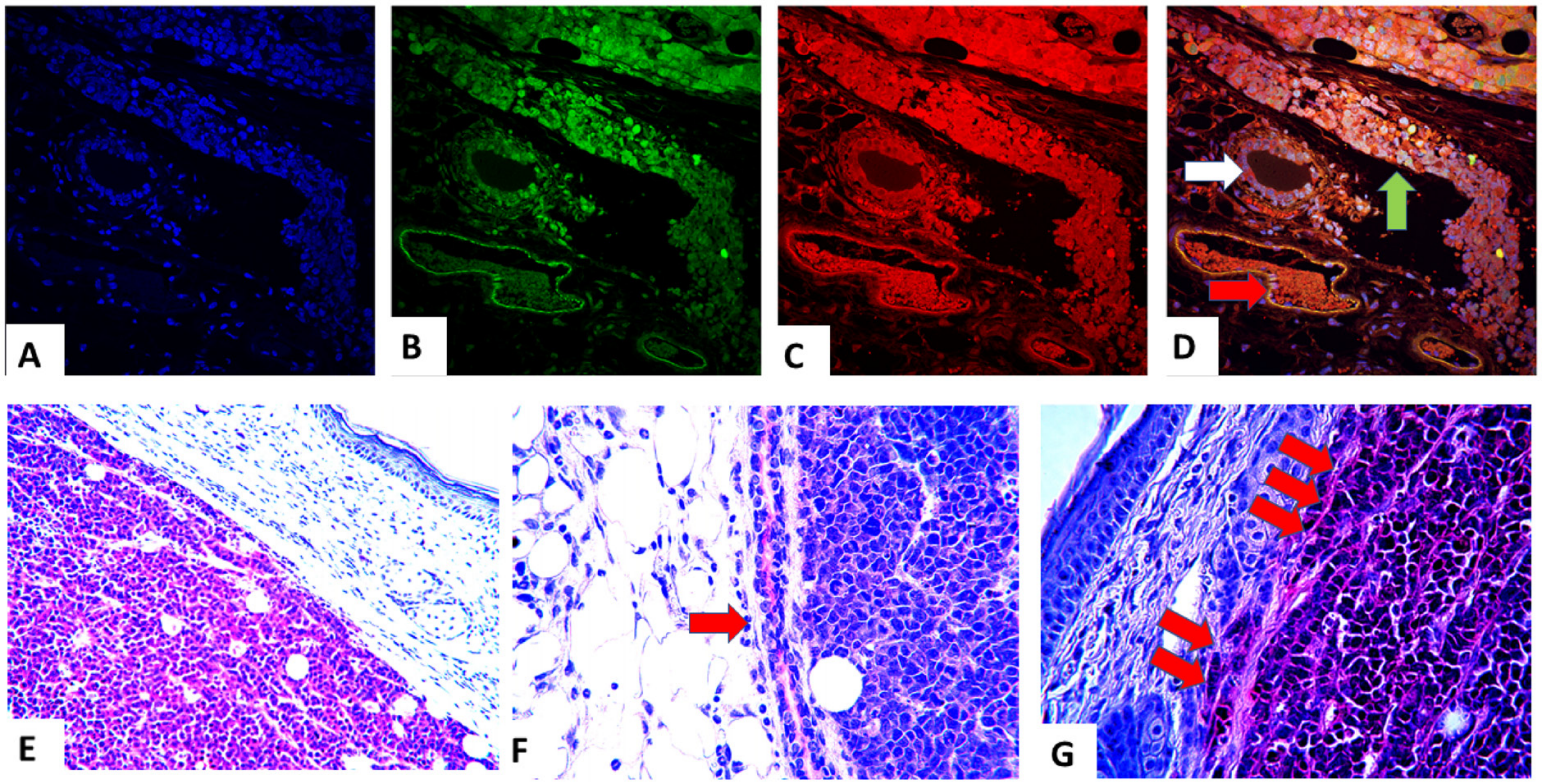
Trifluorescence studies of stages of oncogenesis in the PyVT-iPSC clones. Triple fluorescence studies on extirpated tumors with DAPI blue nuclear autofluorescence (**A**), GFP green autofluorescence (**B**), Alexa Fluor^®^ 594 red immunofluorescence using goat anti-rat added to rat monoclonal to PyVT antigen (**C**) and its merged overlay depicted (**D**). Expression of PyVT is detected not only within the areas of invasive carcinoma (green arrow), but also within the normal breast ducts (white arrow) and breast ducts containing ductal carcinoma *in situ* (DCIS) (red arrow). However, murine angiogenesis (dark areas) did not exhibit any GFP autofluorescence or immunofluorescence (A–D). Colorimetric immunocytochemistry studies utilizing A Fast Red precipitating chromogenic substrate system coupled with alkaline phosphatase conjugated goat anti rat and rat anti-PyVT revealed red chromogenicity not only within the invasive carcinoma (**E**) but also within adjacent normal ducts (red arrow) (**F**) and DCIS areas (double red arrow) (**G**).

Additional detailed analyses of the extirpated PyVT-iPSC tumors (Figure 8A–8D) revealed dual GFP autofluorescence and PyVT cytoplasmic red immunofluorescence not only within the areas of invasive carcinoma (green arrow), but also within the normal breast ducts (white arrow) and breast ducts containing ductal carcinoma *in situ* (DCIS) (red arrow). In contrast, angiogenesis (dark areas) did not exhibit any GFP autofluorescence or immunofluorescence. The injected PyVT-iPSC clones therefore did not differentiate into endothelial cells that resulted in angiogenesis. Angiogenesis occurred from murine precursor cells and not from the injected iPSCs. The same observations were made with injected ErbB2-iPSC cells.

A Fast Red precipitating chromogenic substrate system coupled with alkaline phosphatase conjugated goat anti-rat and rat anti-PyVT revealed red chromogenicity not only within the invasive carcinoma and DCIS areas, but also within adjacent ducts (Figure 8E–8G). Similar results were observed with the ErbB2-iPSC tumors (data not shown).

### RT-PCR, RT-Real time PCR and Western blot studies

Specific studies were carried out to demonstrate and quantitate expression of the oncogenic transgenes, PyVT and ErbB2, in the various cell lines, iPSC clones and emerging tumors. These studies included RT-PCR (Figure 4A), Western blot (Figure 4B) and RT real time PCR (Figure 4C). Only transgenic mammary cancers (and their metastases) and mammary cancers (and their metastases) arising from orthotopically injected transgene-containing iPSC clones expressed the relevant transgene (Figure 4A–4C).

### Quantitative digital image analysis

**Figure 9:**
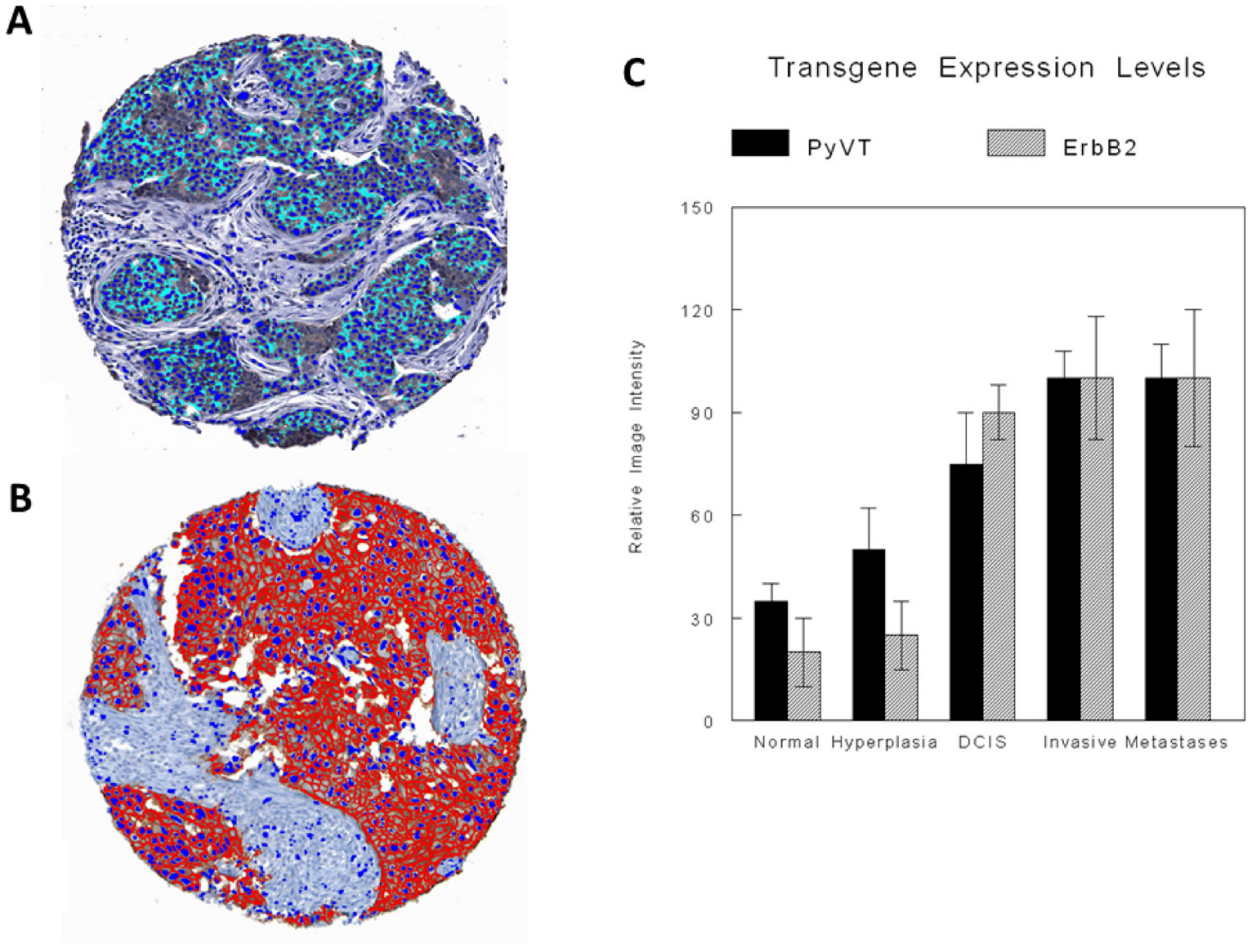
Digital image analysis with specific recognition algorithms (SRAs). Digital image analysis of relative fluorescence and colorimetric immunocytochemistry of representative TMA cores of transgene-iPSC clones illustrate quantitative PyVT cytoplasmic immunoreactivity (**A**) and quantitative ErbB2 membrane immunoreactivity (**B**). Relative expression levels of both transgenes in normal ducts, ducts with hyperplasia, ducts with DCIS and invasive carcinoma and metastatic carcinoma are illustrated in the cases of transgene-iPSC clones. (**C**) Relative expression levels of both transgenes in the tumors arising within the transgenic mice showed similar results.

Digital image analysis was performed on virtual microscopic scanned images from a tissue microarray (TMA) created from the mammary fat pads of different iPSC-transgene-injected groups. Using previously developed epithelial recognition algorithms (ERAs) and specific recognition algorithms (SRAs), which included cytoplasmic and membrane recognition algorithms [28–30] for the PyVT (cytoplasmic signals) (Figure 9A) and the ErbB2 (membrane signals) (Figure 9B) transgenes, quantitation and comparison of relative intensities of the fluorescent and immunocytochemical signals of the respective transgenes were carried out in normal ducts, ducts with hyperplasia, ducts with carcinoma *in situ* (DCIS), and invasive carcinoma. There was a progressive increase in transgene expression in normal, hyperplasia, ductal carcinoma *in situ* (DCIS) and invasive/metastatic cancer with both transgenes (Figure 9C).

**Figure 10:**
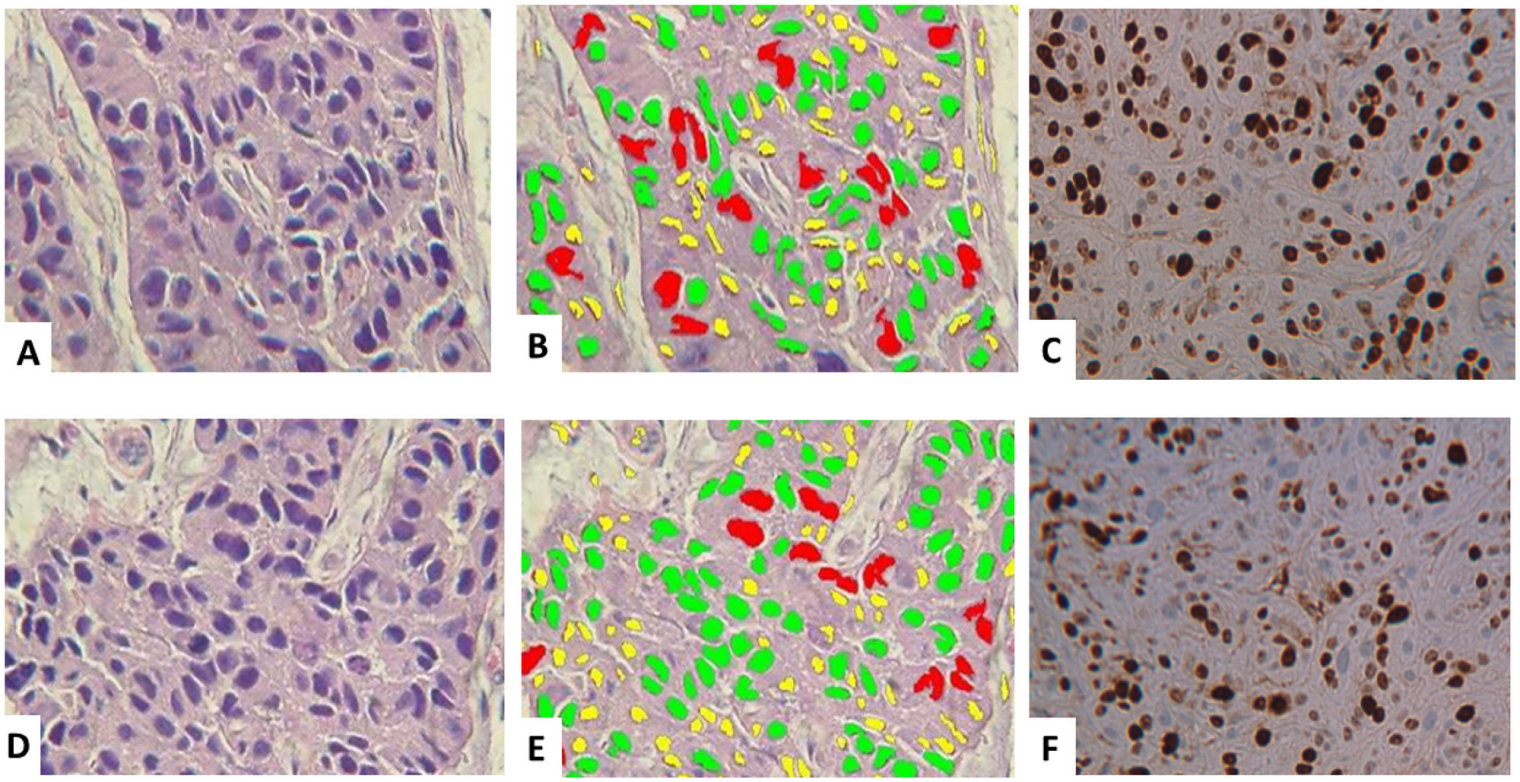
Histology and digital image analysis with additional specific recognition algorithms (SRAs). The tumors derived from the PyVT-containing iPSC clones (**A**–**C**) *vs* the tumors within the PyVT transgenic mice (**D**–**F**). Histology was similar (A), (D). Nuclear size algorithms revealed also revealed a similar spectrum of nuclear sizes ranging from 5–10 μ (yellow), 11–15 μ (green) and 15–25 μ (red) (B), (E). Nuclear algorithms quantitating nuclear Ki-67 immunoreactivity revealed a high proliferation index (> 70%) in both the tumors derived from the PyVT-containing iPSC clones (C) as well as the tumors occurring within the PyVT transgenic mice (F).

Digital image analysis was also used to quantitate tumor grade and confirm the strong histological similarities between the breast cancers arising from the PyVT-iPSC clones (Figure 10A and 10B) and the breast cancers arising within the PyVT transgenics (Figure 10D and 10E). The Ki-67 proliferative index was also similar in both the iPSC tumors (Figure 10C) as well as the transgenic tumors (Figure 10F). The same similarities were observed in tumors arising from the ErbB2-iPSC clones and the tumors arising within the ErbB2 transgenics (data not shown).

## DISCUSSION

Transgenic models of breasts cancer provide powerful models to study breast oncogenesis [21–23]. Actions of multiple breast oncogenes and the molecules they interact with have been elucidated using these models. Interestingly, breast cancers that emerge in most of these models go through the precancerous stages of breast cancer progression before they progress to invasive and metastatic breast cancer. Although many of these models produce multifocal breast cancers, sometimes inaccurately termed polyclonal cancers, the mice harboring the cancers show a normal ductal alveolar system in the adjacent normal breast. This indicates that most of the epithelial cells comprising the mammary ductal lobular system do not transform and therefore mimic, at least in part, the human situation in both spontaneous, sporadic as well as inherited germline breast cancers where only limited foci give rise to cancer. The vast majority of breast cells do not transform and therefore breast cancer is rare at a cellular level

In order to support a possible mechanism related to this observation, we created iPSC clones from tail vein fibroblasts derived from the respective transgenics and non-carrier background mice to see whether we could perturbate their differentiation state both *in vitro* and *in vivo* to eliminate the critical window of differentiation that allows for both breast ontogenesis and breast oncogenesis.

**Figure 11:**
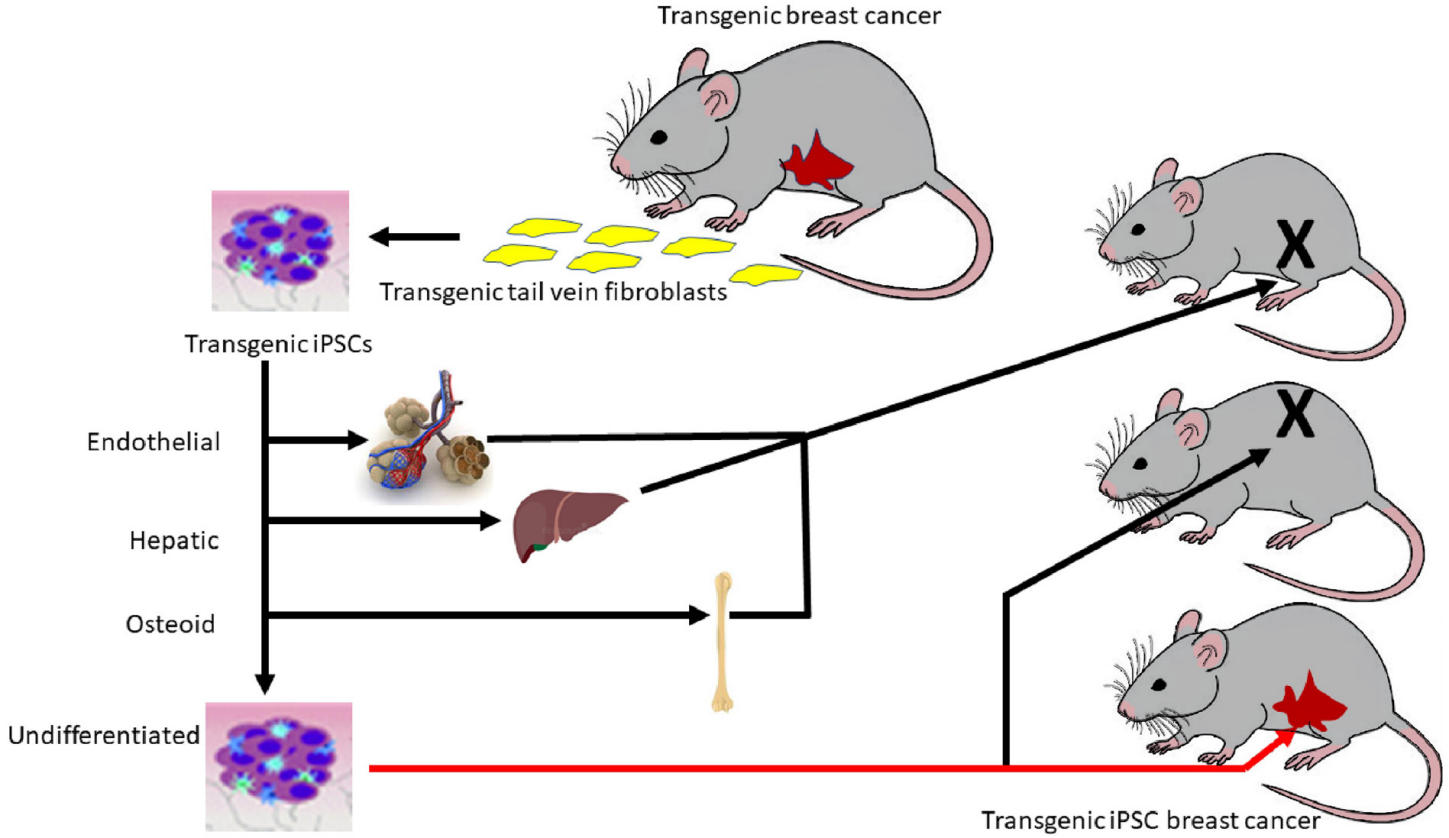
Regulation of oncogenesis by a critical window of differentiation. Schematic depicts the hypothesis that a critical window of differentiation dictates successful breast oncogenesis. The transgenic iPSC clones give rise to breast ontogeny followed by breast oncogenesis only when undifferentiated and injected orthotopically. When differentiated *in vitro* or when injected non-orthotopically, no transformation occurs.

Our findings indicate that both *in vitro* and *in vivo* differentiation regulate breast oncogenesis (Figure 11). If we drive the transgenic iPSC clones to differentiate into endothelial, hepatic or osteogenic lineage directions, not only is the transgene not expressed, but the differentiated clones will not differentiate *in vivo* into breast nor breast cancer when injected into the mammary fat pad. When we inject the undifferentiated iPSC clones into the mammary fat pad, they first probably differentiate into mammary stem cells that then mature into the ductal system and subsequently transform into precancerous and invasive cancerous epithelium. The transgene becomes transformative only when it can act on the iPSCs that have begun to differentiate along a mammary lineage. When the select undifferentiated iPSC clones are injected into a non-orthotopic site, they do not differentiate into a mammary gland nor do they participate in mammary oncogenesis. This observation means two things. First, paracrine factors in the mammary fat pad microenvironment are necessary to induce differentiation of the iPSCs into mammary stem cells capable of further differentiation into the mammary ductal-alveolar system. Second, only after this has happened can transformation occur. Possible transcriptional mechanisms involved in this process have been studied previously [31–34]. At non-orthotopic sites, this induction does not occur. Only when this differentiation occurs, but not before, can breast oncogenesis be initiated. A critical window of differentiation must exist before breast oncogenesis can commence. The transgene drives this oncogenesis and its expression increases with the progressive stages of breast cancer progression. iPS-PyVT cells probably first differentiate into mammary stem cells under the paracrine influence of mammary fat pad microenvironment and then mature into breast ducts that then transform under the influence of the oncogenic transgene into precancerous and then invasive breast cancer, mirroring the same sequence observed in the FVB/N-Tg (MMTV-PyVT)634Mul/J and FVB-Tg (MMTV-ErbB2) NK1Mul/J transgenics. The strong resemblance of histology, tumor grade and Ki-67 immunoreactivities between the tumors arising from the PyVT-iPSC clones and the tumors arising within the PyVT transgenics suggest that we have successfully used the iPSC clones as transgenic surrogates. To our knowledge no other study has utilized this transgenic oncogene-iPSC clone approach. These iPSC clone surrogates allow us to further examine our “critical window of differentiation” hypothesis to explain the rareness of transformation at a cellular level.

We certainly do not dispute the requirement of transgenes (oncogenes) and stem like progenitor cells in breast oncogenesis but the simple fact remains that while there are many stem like progenitor cells containing the necessary transgenes (oncogenes), only a small minority of cells actually transforms. Our study notes this, offers an hypothesis. and provides initial supporting data. We do acknowledge, however, that there could be other viable hypotheses to explain our observations.

Although other investigators have demonstrated that a single murine mammary stem cell, when injected into the cleared mammary fat pad, is sufficient to generate an entire mammary ductal tree [35, 36] and that murine embryonic stem cells, when induced to undergo hematopoietic differentiation *in vitro* and then injected into the mammary fat pad, are able to exhibit mammary morphogenesis [37], we believe that our study is the first to observe both mammary ontogenesis as well as oncogenesis.

Our iPSC transgene model further allows for both *in vitro,* as well as *in vivo* dissection of those factors, that may more precisely define the critical window of differentiation. Using the many different iPSC clones that we have created from the oncogenic transgenics, we can experiment with a various number of differentiating agents attempting to induce a mammary lineage differentiation, something that has not been done previously from oncogene-containing iPSC clones derived from adult fibroblasts. To date and to the best of our knowledge, no one has successfully driven iPSCs or oncogene-containing iPSC clones derived from adult fibroblasts into mammary gland differentiation *in vitro*, though both iPSCs and embryonic stem cells, using their derived embryoid bodies (EBs), have been differentiated into hepatocyte, hematopoietic, osteogenic and endothelial lineages *in vitro* using cocktails of defined cytokines and growth factors [38–42]. Murine embryonic stem cells (mES), but not iPSCs, did differentiate in 3D Matrigel chambers into ductal-alveolar structures that expressed ductal epithelial and myoepithelial markers. However, they were negative for secretory markers of β-casein and whey acidic protein (WAP). More recent studies have successfully induced mammary gland differentiation *in vitro* but by only first overcoming lineage-specific restrictions on differentiation by either co-culture experimentation or using non-neural ectoderm or epithelial cells rather than adult fibroblasts as starting material [43–45]. None of these studies have used oncogene-containing iPSC clones derived from any source. As mentioned previously, mES, when differentiated *in vitro* and then injected, exhibited mammary morphogenesis [37]. Obviously, the mammary microenvironment in both situations plays a key role in mammary morphogenesis. Still, from the mammary epithelial perspective, regulatory networks orchestrated by key transcription factors (TFs) also play a role in mammary differentiation. In mES, a set of core TFs, notably Oct4, Sox2 and Nanog, form an autoregulatory network and act cooperatively to activate genes capable of maintaining the embryonic stem cell state and, at the same time, silence the expression of genes involved in lineage-specific differentiation [31, 33, 46]. It has been shown that Slug and Sox9 also act cooperatively to regulate the mammary stem cell state [32]. If we can more precisely differentiate our transgene-iPSC clones into mammary gland differentiation *in vitro*, then we can derive sorted subpopulations of differentiating cells to determine at what point *in vivo* ontogenesis and oncogenesis is enhanced or lost. We could then select the injected iPSC clones that show the most robust mammary gland morphogenesis and that exhibit differentiation along all three mammary gland lineages: luminal cells, secretory cells and myoepithelial cells *in vitro* to use our model in a reverse direction.

iPSC clones that show the most promise in terms of mammary gland development can then be harvested from the fat pads by collagenase, dispase and trypsin digestion over a time course to monitor lineage-specific differentiation. We could gate and sort the retrieved cells based initially on GFP. According to the epithelial differentiation hierarchy model [47], mouse mammary glands consist of a hierarchy of several cell types. These include multipotent stem cells which can regenerate entire mammary glands in mice at the single cell level, bipotent progenitor, unipotent progenitor and differentiated cells, which are delineated by different combinations of cell surface markers. These cell surface markers and fluorescence-activated cell sorting (FACS) could be further used to analyze the cells and divide them into the following fractions: multipotent stem cells (CD29hiCD49hi CD24+ESA-), luminal progenitor (CD61+CD49loESA+CD24+CD29lo) and ductal cells (CD24+CD61-CD29lo), as well as parity-induced mouse mammary epithelial (PI-MEC) cells (CD24hiCD49lo).

Some of these cells, eg., luminal progenitor cells, have been thought to be the targets of ErbB2 gene-induced tumorigenesis [47, 48]. Other investigators have challenged these observations, arguing that PI-MEC cells are the true targets of ErbB2 tumorigenesis [49]. Certainly, more insights are needed using our iPSC transgenic model. One basic question we hope to answer is the time course of oncogene expression. PyVT or ErbB2 expression did not occur in the derived iPSC clones *in vitro* nor was it induced by dexamethasone, even though at least theoretically, a dexamethasone responsive MMTV promoter lies upstream of the transgene. What this means is that there are other *in vivo* mammary factors required to stimulate MMTV-transgene expression.

Oncogene-induced transformation requires not only the expression of the oncogene but also the activation of oncogene-mediated pathways. Therefore, our transgene iPSC model should allow us to investigate in which subpopulation (s) and at what time point (s) the oncogene-mediated pathway (s) are activated. The two oncogenes, PyVT and ErbB2, activate very similar pathways involving cellular kinases and phosphatases [50–53], recruitment of activated c-Src, activation of Ras/Erk and PI3K/Akt signaling [21]. Integration of these multiple pathways ultimately induces cellular transformation. Therefore, it is reasonable to hypothesize that outside this critical window of differentiation all the pathways activated by the oncogene are neutralized by events related to the early and late stages of differentiation. We could then study gene expression patterns of the oncogene-activated pathways in the unsorted and sorted subpopulations over the time course of mammary ontogenesis and oncogenesis. Another basic question in mammary oncogenesis derived from the transgene-containing iPSC clones is the expression of genes responsible for iPSC induction, e. g. Sox2, Oct3/4, Klf4 and Myc. Certainly, one would expect that the expression of these genes might decrease with mammary ontogenesis and oncogenesis. As far as mammary oncogenesis is concerned, it has also been shown that ErbB2 and PyVT may regulate cancer stem cells and cancer stem cell pathways [50–53]. Therefore, a study of expression patterns of the four key genes used for iPSC induction, more global transcriptome profiling and additional microarray analysis on these same subpopulations might shed insight into the critical window of differentiation.

## MATERIALS AND METHODS

### Generation of iPSC clones

Tail vein fibroblasts were isolated as discussed under “Animal studies” and prepared for retroviral transfection. A Platinum-A Retroviral Packaging Cell Line, Amphotropic (Cell Biolabs, San Diego, CA, USA) was prepared. A Murine Stem Cell Factor Retroviral Vector Set (4 Genes) with pMX-GFP Retroviral Vector (Cell Biolabs) was obtained and each plasmid was separately transfected according to the manufacturer’s instructions with vectors: pMX-GFP, pMXs-mOct3/4, pMXs-mSox2, pMXs-mc-Myc and pMXs-mKlf4. For each type of transduction, 2.0 × 10^5^ tail vein fibroblasts were plated in a 60 mm culture dish in complete culture medium (DMEM with high glucose, 10% FBS, 1% PS), washed and transduced drop- wise with a mixture of vector-containing supernatant filtered through a 45 um cellulose acetate syringe filter (Whatman, Tisch Scientific, North Bend, OH, USA). The process was repeated.

### Identification and selection of iPSC clones

After two cycles of retroviral transduction, the transduced fibroblasts were cultured in embryonic stem cell (ES) medium (DMEM containing 15% FBS (vol/vol), 2 mM L-GIn, 1 × 10^-4^ M nonessential amino acids, 1 × 10^-4^ M 2-mercaptoethanol, 10 ng/mL LIF and 50 mg/mL of penicillin and streptomycin). Each day, the fibroblasts were fed with fresh ES medium to generate iPSC clones. Colonies became visible approximately 8 days after the retroviral infection in transduced fibroblasts from each group. The morphology of many of the iPSC clones were similar and typical of iPSC clones. However, some of the clones appeared more fibroblastic and more rapidly growing. Around 15 days after retroviral transduction, representative iPSC clones were picked and cultured in 24-well plates seeded with SNL feeder cells [54].

### Alkaline phosphate staining

The culture medium was aspirated, and cells were washed twice with 2 mL of PBS. The cells were fixed with 0.5 mL of 4% paraformaldehyde in PBS for 1–2 minutes. 2 mL of Fix Solution was added, and cells were incubated at room temperature for 1 to 2 minutes. 1 mL of freshly prepared AP Staining Solution was then added.

### iPSC marker immunofluorescence

The iPSC clones were confirmed as IPSCs with a battery of rabbit anti-mouse IPSC markers including Oct3/4, Sox2, c-Myc, mKlf4, Nestin and SSEA-1 (Thermo Fisher Scientific, Waltham, MA, USA). The secondary antibody was an Alexa Fluor 594-conjugated goat anti-rabbit (Thermo Fisher Scientific), all used in accordance with the manufacturer’s conditions. To immobilize the iPSC clones, glass-bottom dishes were coated with Cell-TEK adhesive. The adherent iPSC was then fixed with 4% paraformaldehyde, after permeabilizing with TX-100 and blocking with normal goat serum. The iPSC clones were then incubated with the primary antibodies, washed, and incubated with the secondary antibodies. The dishes were finally mounted with Vectorshield mounting medium with DAPI (#H-1200) (Vector Laboratories, Burlingame, CA, USA) and viewed with an Olympus Fluoview-1000 confocal scanning system under different wavelengths.

### RT-PCR, RT-Real time PCR and Western blot studies

These studies were used to investigate the iPSC clones for expression of the relevant oncogenic transgenes. They were also used to evaluate expression of the transgenes within the emerging murine tumors as discussed in the section, “Animal studies.”

Freshly picked iPSC clones and clones that were frozen and quickly thawed, as well as extirpated tumors described under “Animal studies,” were made available for study. RNA quality and quantity were determined by measuring absorbance at 260 and 280 nm. Oligo (dT) primers (Integrated DNA Technologies, Coralville, IA, USA) were used with Superscript II reverse transcriptase (Invitrogen, Waltham, MA, USA) for cDNA synthesis from 1 μg total RNA extracted from the transgenic-iPSC clones and the non-carrier iPSC clones. PCR was conducted with Taq Polymerase (Invitrogen, Inc.). The primer sequences used (Invitrogen, Inc., Carlsbad, CA, USA) for RT-PCR of PvVT were:

5′-CTTCCAGAADGGCGGAGCGAG-3′ Forward, 5′-CCCGAAGAATCAGACCCTCCC -3′ Reverse, which produced a 500 bp product. Since the PyVT gene is alternately spliced, primers that eliminated the contribution of genomic DNA were selected. The primer sequences (Invitrogen) used for RT-PCR of ErbB2 were: 5′-AGTGTGTACCGGCACAGACAT-3′ Forward and 5′-TGTCACTGGTGATGGCCCTCG-3′ Reverse, which produced a 500 bp product. The mixture was first heated at 94° for 2 min in a PTC-200 DNA Engine Thermal Cycler (Bio-Rad Laboratories, Hercules, CA, USA). Amplification was performed for 33 cycles at 94° for 30 s, 55° for 30 s and 68° for 60 s, followed by 72° for 10 min. The PCR products were separated by electrophoresis on 1.5% agarose gels, premixed with ethidium bromide at concentrations of 0.1–0.5 ug/ml. Digital images were captured on a Fotodyne gel documentation system.

For western blot studies, fresh and/or frozen material was quick thawed and made available for study. Cells and extirpated tumors were extracted with a buffer (1% Triton X-100, 150 mM NaCl, 10 mM sodium phosphate, 10 mM EDTA). The samples were then centrifuged at 13,000 *g* at 4°C for 15 min. Protein concentrations were determined using the BCA protein assay (Pierce Biotechnology, Rockford, IL, USA). Samples containing equal protein were boiled in Laemmli buffer under reducing conditions, run on a 4–15% SDS-polyacrylamide gel, and transferred to a PVDF membrane that was then incubated with the respective primary antibody, (rat anti-PyVT (Santa Cruz Biotechnology, Santa Cruz, CA, USA), rabbit anti-ErbB2 (Thermo Fisher Scientific), HRP-labeled goat anti-rat, or goat anti-rabbit IgG (Cell Signaling Technology, Danvers, MA, USA). Bound antibodies were detected by a chemiluminescent detection system (West Femto) (Pierce Biotechnology) according to the manufacturer’s instructions, or with the Supersignal West Dura extended Duration Substrate (Pierce Biotechnology). A monoclonal antibody to Glyceraldehyde 3-phosphate Dehydrogenase (GAPDH) (Santa Cruz Biotechnology) was used to normalize protein loading. A CCD Imaging system, the ChemiDoc™ MP Imaging System (Bio Rad, Hercules, CA, USA) was used to quantitate the signal.

RT-real time PCR was also performed. For real-time PCR, reactions were run on a 7500 Real-Time PCR system (Applied Biosystems, Inc., Foster City, CA, USA). Gene expression was detected with the SYBR Green Master Mix. Relative gene expression was determined by normalizing to β-actin using the Δ C _T_ method with 7500 System SDS software (Applied Biosystems, Foster City, CA, USA). Primers used for real time RT-PCR (Invitrogen) for PyVT were as follows: 5′-TTTGGAACACCAACCCGAGA-3′ Forward and ATCCAGGTCCAGCCAGTCTAT-3′ Reverse and primers used for ErbB2 were: 5′-ATTGGCTCTGATTCACCGCA-3′ Forward and 5′-CAAGCCCTCGAGACCACAAT-3′ Reverse. The primers used for the analysis of β-actin were: 5′-GGCACCCAGCACAATGAAG-3′ Forward, 5′-GCCGATCCACACGGAGTACT-3′ Reverse. An additional housekeeping control was used, GAPDH. The primers used for the analysis of GAPDH were: 5′- ATGGGGAAGGTGAAGGTCG-3′ Forward, 5′- GGGGTCATTGATGGCAACAATA-3′ Reverse.

### Pluripotent differentiation of iPSC clones

#### Differentiation studies

Pluripotent differentiation was carried out along three different lineages: endothelial, hepatic and osteogenic. Each lineage differentiation strategy involved two steps, formation of embryoid bodies and subsequent lineage differentiation. As the differentiation process was occurring, the cells in each group were monitored by phase contrast microscopy.

#### Biomarker studies

For each of the lineages, proof of successful differentiation was obtained by the detection of specific biomarkers for each lineage: CD31 for endothelial, albumin for hepatic and osteocalcin for osteogenic. Cultures of the differentiated cells that grew as monolayers were subjected to double immunofluorescent studies using the following combinations of antibodies: rabbit anti-mouse CD31, rabbit anti-mouse albumin, rabbit anti-mouse osteocalcin followed by Alexa Fluor 594-conjugated goat anti-rabbit (all antibodies from Abcam, Cambridge, MA, USA). The adherent monolayers were then fixed with 4% paraformaldehyde, after permeabilization with TX-100 and blocking with normal goat serum. The spheroids were then incubated with the respective primary antibodies according to the manufacturer’s specifications, washed with PBS 4–5 times, and incubated with the secondary antibody again according to the manufacturer’s specifications. The dishes were finally mounted with Vectorshield mounting medium with DAPI (#H-1200) (Vector Laboratories) and viewed with an Olympus Fluoview-1000 confocal scanning system.

### Animal studies

Ten each of 4-week-old female FVB/N-Tg (MMTV-PyVT)634Mul/J and FVB-Tg (MMTV-ErbB2) NK1Mul/J and 50 noncarrier FVB background mice were obtained (The Jackson Laboratory Biomedical Research Institute, Bar Harbor, ME, USA).

#### Tail vein procurement studies

Tail vein fibroblasts from each group were isolated by cutting 5 cm of tail from 2-month-old mice, peeling the dermis and mincing the tail tips into 1 cm pieces. A pair of pieces was plated on a 600 mm collagen I coated dish (BD Biosciences, Bedford, MA, USA) with 5 mL DMEM containing 10% FBS (Sigma-Aldrich, St. Louis, MO, USA). After 5 days of incubation, fibroblasts migrated out of the tail pieces and were transferred to new dishes and allowed to proliferate.

#### Implantation and harvesting studies

Clones were injected into the cleared inguinal mammary fat pads of background FVB mice. The mammary fat pads were previously cleared at 3–4 weeks of age according to accepted procedures [55]. To test the tumorigenesis of the iPSC clones, three types of cells were implanted: 1) Noncarrier iPSC clones; 2) iPSC clones from FVB/N-Tg (MMTV-PyVT)634Mul/J; and 3) iPSC clones from FVB-Tg (MMTV-ErbB2) NK1Mul/J. The iPSC clones obtained from each group that were subjected to *in vitro* differentiation were also similarly injected. iPSC clones of all the groups were also implanted non-orthotopically in areas such as the flank and back. Approximately, 5 × 10^5^ cells were used for each injection. The mice were observed for the next 8–16 weeks. In some mice emerging tumors were obvious. In any case after this period, the mammary fat pad and the non-orthotopic sites were extirpated. Extirpated areas were either snap frozen or immediately processed for RT-PCR, Western blot and RT-real time PCR analysis and routine light microscopy, trifocal immunofluorescence and digital image analysis.

#### RT-PCR, RT-Real time PCR and Western blot studies

These studies have been enumerated previously under the section, “Identification and Selection of iPSC Clones.”

#### Histological studies

Fresh-frozen and paraffin-embedded tissues of the extirpated tissues including tumors were processed according to standard protocols involving dehydration, paraffin embedding, sectioning and staining with hematoxylin and eosin as well as cutting sections that were left unstained.

#### Fluorescence and immunocytochemical studies

Sections of the extirpated tissues and tumors were then treated by target antigen retrieval solution (DAKO, Carpinteria, CA, USA) in a steamer for 40 min and allowed to cool for 20 min and rinsed in PBS. After treatment with 0.1% Triton X-100 in PBS for 5 min, tissue sections were incubated with 5% normal donkey serum in PBS for 1 hour followed by incubation of primary antibodies, rat anti-PyVT (Santa Cruz Biotechnology), rabbit anti-ErbB2 (Thermo Fisher Scientific) and rat anti-mouse Ki-67 (DakoCytomation, Carpinteria, CA, USA). Tissue sections were then washed three times in PBS for 5 min each and incubated with the appropriate secondary antibodies of either Alexa Fluor 594-conjugated goat anti-rat or Alexa Fluor 594-conjugated goat anti rabbit (all antibodies from Thermo Fisher Scientific) for the triple fluorescence studies or an alkaline phosphatase-conjugated goat anti-rat (Sigma-Aldrich Chemicals, St. Louis, MO, USA) or alkaline phosphatase-conjugated goat anti-rabbit (Abcam) or biotinylated polyclonal rabbit anti-rat followed by peroxidase-conjugated streptavidin (DakoCytomation) for the immunocytochemical studies. The color was developed with A Fast Red precipitating chromogenic substrate system or 3,3′-diaminobenzidine tetrahydrochloride. For these immunocytochemical studies, the slides were countered stained with hematoxylin. For the fluorescence studies, the sections were finally mounted with a Vectorshield mounting medium with DAPI (Vector Laboratories) and viewed with a Olympus Fluoview-1000 confocal scanning system. For the immunocytochemical studies, the slides were viewed with an Olympus microscope with attached digital camera.

### Quantitative digital image analysis

Multiple 2 mm tissue cores of tumor from each paraffin embedded donor block were arrayed into a new recipient paraffin block.

Our specific TMA algorithms took a whole virtual TMA and carried out virtual alignment and core indexing. In this manner a virtual TMA could be created consisting of perfectly oriented horizontal and vertical linear arrays of tissue cores. The process of scanning each TMA slide into a virtual slide took approximately 30 minutes. Subsequent virtual alignment, image processing, and the application of the ERAs and SRAs took an additional 30 minutes/TMA.

Image acquisition by either the Aperio ScanScope T2 System (Aperio, Vista, CA, USA) or the iSCAN System (BioImagene, Inc, Cupertino, CA, USA) produced equivalent results with uniformly produced sharp images with high contrast. For approximately 5% of the acquired images was image quality below the standard where the algorithms were interpretative. These images had to be discarded. For approximately 10% of the images, mask removal and contrast enhancement improved image quality.

ERAs applied to each TMA core were successful in recognizing epithelium, filtering out stroma and determining its epithelial percentage and therefore its cancer cell density. Specific immunoreactivity was then analyzed by the application of the appropriate SRAs which included nuclear, cytoplasmic and membrane recognition algorithms. The ability of the algorithms to recognize the cellular compartments of the normal, precancer and cancer cell, calculating the cross-sectional area of the cancer cell’s nucleus as a determinant of tumor grade and detect the appropriate immunoreactivity which was present and quantitate it on both ordinal as well as continuous scales has been demonstrated previously [28–30]. The algorithm-based determination of immunoreactivity was the same every time the algorithm was run and therefore showed no interobserver, intraobserver or fatigue variability.

### Statistical analysis

All experiments were performed with a minimum of three replicates and representative results depicted. Declarations of differences imply differences of statistical significance. Significance was assessed by the Student’s *t*-test.

## Abbreviations

iPSCs: induced pluripotent stem cells
MMTV: mouse mammary tumor virus
PyVT: polyomavirus middle T antigen
SSEA-1: mouse-specific stage-specific embryonic antigen-1
ERAs: epithelial recognition algorithms
SRAs: specific recognition algorithms
GFP: green fluorescence protein
DCIS: ductal carcinoma *in situ*
TMA: tissue microarray
WAP: whey acidic protein
FACS: fluorescence-activated cell sorting
TFs: transcription factors
PI-MECs: parity-induced mouse mammary epithelial cells
mES: murine embryonic stem cells
